# Observations of sharks (Elasmobranchii) at Europa Island, a remote marine protected area important for shark conservation in the southern Mozambique Channel

**DOI:** 10.1371/journal.pone.0253867

**Published:** 2021-10-05

**Authors:** Mireille M. M. Guillaume, Bernard Séret

**Affiliations:** 1 Laboratoire BOrEA MNHN-SU-CNRS-IRD-UCN-UA EcoFunc, Aviv, Muséum National d’Histoire Naturelle, Paris, France; 2 Laboratoire d’Excellence CORAIL, Perpignan, France; 3 ICHTYO-CONSULT, Igny, France; Wildlife Conservation Society Canada, CANADA

## Abstract

Sharks have declined worldwide and remote sanctuaries are becoming crucial for shark conservation. The southwest Indian Ocean is a hotspot of both terrestrial and marine biodiversity mostly impacted by anthropogenic damage. Sharks were observed during surveys performed from April to June 2013 in the virtually pristine coral reefs around Europa Island, a remote Marine Protected Area located in the southern Mozambique Channel. Observation events comprised 67 1-hour scientific dives between 5 – 35m depth and 7 snorkeling inspections, as well as 4 dinghy-based observations in the shallow lagoon. In a period of 24 days, 475 sharks were tallied. *Carcharhinus galapagensis* was most encountered and contributed 20% of the abundance during diving, followed by *C*. *albimarginatus* (10%). Both species were more abundant between 11-14h, and on the exposed sides of the island. Numbers of *Sphyrna lewini* were highest with 370 individuals windward and leeward, mostly schooling. *S*. *lewini* aggregations in the area are hypothesized to be attracted to the seamount archipelago offering favorable conditions for deep incursions and of which Europa Island forms part. *C*. *amblyrhynchos*, *Galeocerdo cuvier* and *S*. *mokarran* were uncommon, while there was an additional observation of *Rhincodon typus*. The lagoon of Europa was a nursery ground for *C*. *melanopterus* where it was the only species present. A total of 8 species was recorded, contributing to the shark diversity of 15 species reported from Europa since 1952 in the scientific and gray literature. Overall, with the occurrence of several species of apex predators in addition to that of *R*. *typus*, large schools of *S*. *lewini*, fair numbers of reef sharks and a nursery of *C*. *melanopterus*, Europa’s sharks constitute a significant reservoir of biodiversity, which contributes to preserve the functioning of the ecosystem. Our observations highlight the relevance of Europa Island for shark conservation and the need for shark-targeted management in the EEZ of both Europa and Bassas da India.

## Introduction

Sharks as predators are a major component of marine food webs [1, 2]. Among them, the larger species, such as the great and the scalloped hammerhead sharks *Sphyrna mokarran* and *S*. *lewini* and the tiger shark, *Galeocerdo cuvier*, are transient apex predators on coral reefs [2, 3]. They are capable of structuring coral reef food webs both directly through predation and indirectly by modifying prey behavior [3]. In contrast, most shark species, among which the gray reef shark, *Carcharhinus amblyrhynchos*, the Galapagos shark, *C*. *galapagensis*, the blacktip reef shark, *C*. *melanopterus*, are reef-associated meso-predators [2, 3]. They share functional redundancy among each other and with large piscivorous fishes such as groupers or snappers; all are assigned to the trophic group of high-level meso-predators and are considered to exert a limited influence on the community structure [2–4].

Due to overfishing, shark abundance steeply declined worldwide over the past five decades [5–10], rendering remote areas critical for shark conservation. In the Pacific, for instance, the abundance and size of large apex predators represented 54% of the total fish biomass in the remote and lightly fished area of the Northwest Hawaiian Islands, but less than 3% in the urbanized and heavily fished Main Hawaiian islands [11]. Similar biomass proportions were found between Palmyra, with limited fishing pressure, and Christmas and Fanning islands both with extensive shark fisheries [12]. In the Indian Ocean, three decades of poaching by illegal vessels induced collapse by greater than 90% of shark populations in the remote expanses of the Chagos Archipelago [6].

High natural diversity and abundance of sharks are vulnerable to even light fishing pressure [5]. The distance to human influence was shown to be the key factor for shark conservation [13], yet this feature can be mitigated by low fuel cost and rising market price of fish [14]. Compared to fisheries closures, biomass in isolated areas is described by a much higher baseline [15–18]. Indeed, remote areas host far higher biomass of top predators and fish functional diversity than marine reserves [19, 20], and wilderness areas are now the last refuges for the most vulnerable functional roles in marine ecosystems [20, 21]. Such remote areas are therefore crucial for shark conservation and essential for preserving the integrity of marine food webs [22, 23].

Europa Island is the southernmost of the remote *Iles Eparses*, small islands named for being scattered in the southwestern Indian Ocean region. These islands are under French jurisdiction and, since 1973, military control. Europa has been a protected area and a Natural Reserve since 1975, presently managed by the administration of the *Terres Australes et Antarctiques Françaises* (TAAF). Since 1994 only some pelagic tuna fisheries have been permitted under license. Relatively preserved from direct human impacts, this Marine Protected Area is mainly subject to climate change. However, despite the recognition of Europa as a preserved natural environment and the important conservation efforts for the island, including a strict ban on near-shore fishing, poaching does occur. Indeed, Probst [24] and Russel & Ruffino [25] reported catches of sharks and other fishes in the lagoon by resident soldiers and constables (up to 20 sharks were caught in two months, *ibid*.). Also poaching practiced by fishermen of neighboring and some Asian countries has been observed (Jean-Bernard Galvès, pers. comm.).

Sharks around Europa were first studied in 1952 by Fourmanoir [26] who evaluated the potential for a small-scale fishery, including a drying station, but this was not developed [27]. Fourmanoir (*ibid*.) mentioned the presence of just three species, the dusky shark, *C*. *obscurus*, *C*. *melanopterus* and *G*. *cuvier*. In 1961, Fourmanoir [28] added two species: the silvertip shark, *C*. *albimarginatus*, and the spinner shark, *C*. *brevipinna* (under the name *C*. *johnsoni*). Maugé [29] compiled the previous records made by Fourmanoir, and incorrectly denied the occurrence of *C*. *obscurus* in Europa. Probst [24] provided an annotated list of sharks observed in Europa: numerous *C*. *melanopterus*, three *C*. *albimaginatus*, one oceanic whitetip shark, *C*. *longimanus*, two *G*. *cuvier*, and one sicklefin lemon shark, *Negaprion acutidens*. Bertrand *et al*. [30] organized observations made during the production of a wildlife documentary [31]; beside the previously recorded species, they observed two *C*. *amblyrhynchos*, one *C*. sp. cf. *obscurus*, one smalltooth sandtiger shark, *Odontaspis ferox* and numerous *S*. *lewini*. Bourjea *et al*. [32] incorrectly reported *C*. *leucas* (Jérôme Bourjea, pers. comm.), shark that was identified as *Negaprion acutidens* on photography (Jean-Bernard Galvès, pers. comm.). In its survey report, the SOS Foundation [33] recorded 9 species: *C*. *galapagensis*, *C*. *melanopterus*, *C*. *amblyrhynchos*, *C*. *albimarginatus*, the silky shark, *C*. *falciformis*, *G*. *cuvier*, *S*. *lewini*, *N*. *acutidens*, and the white tip reef shark, *Triaenodon obesus*. Séret e*t al*. [34] observed only a few young *C*. *melanopterus*, in the mangrove area and spotted one *S*. *lewini*, and one *C*. *albimaginatus*. Clarke *et al*. [35] using underwater visual surveys (UVS) and baited remote underwater video-camera system (BRUVs) recorded the following sharks at Europa: 55 hammerhead sharks, *S*. *lewini* and *S*. *mokarran*, 17 *C*. *galapagensis*, 14 *C*. *albimarginatus*, and 5 *C*. *melanopterus*. Fricke *et al*. [36] compiled previous records of *C*. *melanopterus*, *C*. *obscurus*, *G*. *cuvier*, plus *C*. *albimarginatus*, *C*. *amblyrhynchos*, *T*. *obesus*, *S*. *lewini*, and *S*. *mokarran* that were incorrectly stated as new records. Chabanet *et al*. [37] mentioned the passage of individuals of *C*. *albimarginatus* and *S*. *lewini* during visual reef census using scuba, observations that were already listed in [36]. These previous records are compiled in Table 2.

The present paper reports on underwater observations of sharks made during the two legs of an expedition during the period from April to June 2013 to survey the coral reefs around Europa. The aim of this paper is to draw attention on the importance of shark populations at this remote island and to provide data to support their conservation in this small area in the ongoing context of the creation of the National Natural Reserve of Europa.

## Materials and methods

### Study area: Europa Island

**Fig 1:**
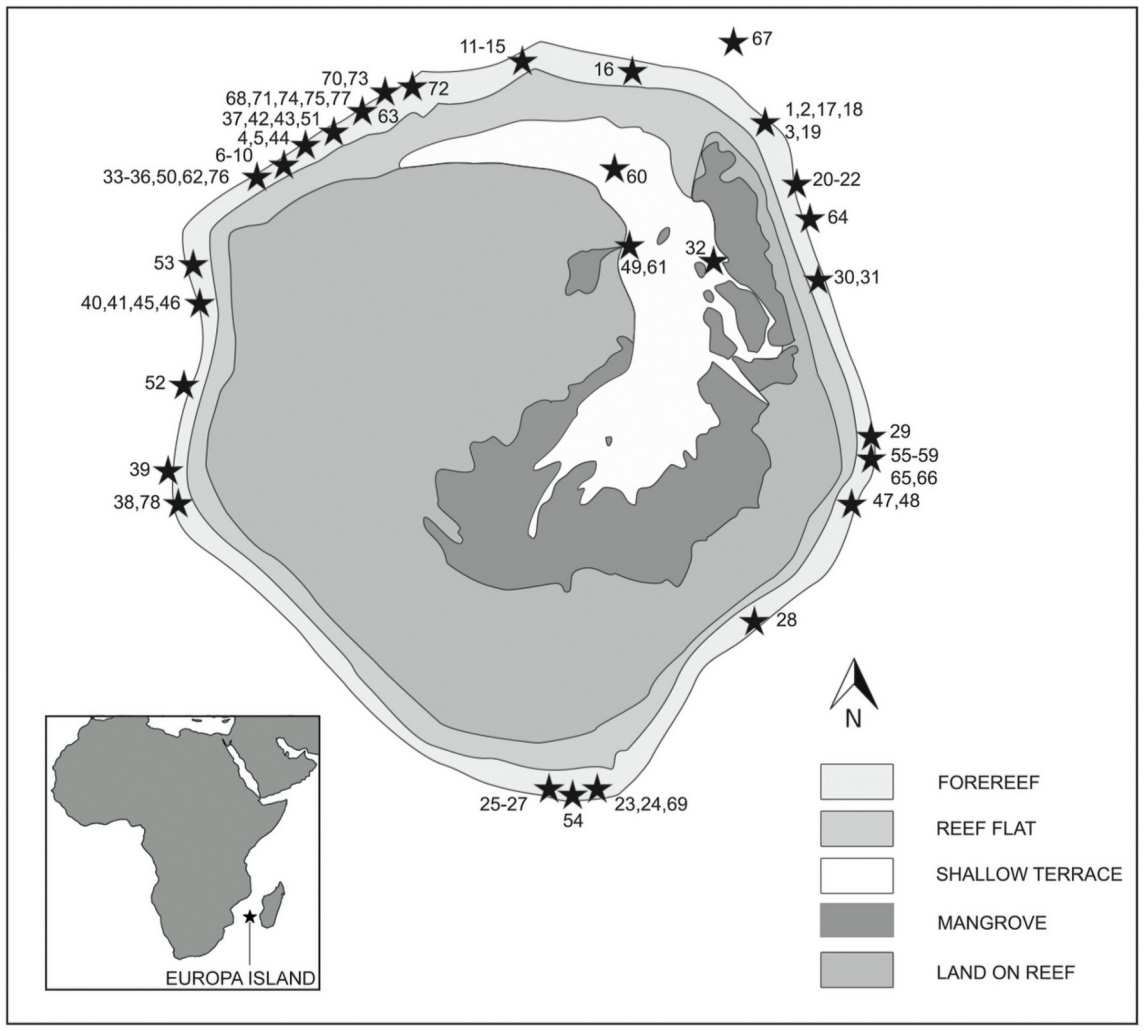
Europa Island. Site location on the forereef and in the lagoon, adapted from https://eol.jsc.nasa.gov/SearchPhotos/photo.pl?mission=ISS005&roll=E&frame=9408 using Adobe Illustrator. Inset shows the position of Europa between Africa and Madagascar, composed using QGIS (version 3.4.6 Madeira).

Europa Island (hereafter Europa), is a small oceanic low island located in the Mozambique Channel, 500 km east of southern Mozambique and 300 km west of southern Madagascar (22°21’ S, 40°21’ E; inset Fig 1). Geologically, Europa, together with Hall Tablemount, Jaguar Seamount, and Bassas da India Atoll, constitutes an archipelago [38 modified] of a cluster of seamounts [39] of volcanic origin aged to ~55 Ma [38, 40]. From a geopolitical point of view, Europa is one of the five *Eparses* Islands belonging to France. Europa’s EEZ covers 127,300 km^2^.

Europa is a small sub-circular atoll of 6–7 km diameter with a total land surface of 28 km^2^. A narrow and shallow lagoon of about 7 km^2^ extends far inland and opens to the sea over the northern reef flat ([41], Fig 1). Saltwater mangrove formations dominate the lagoon’s shoreline [42], while sandy beaches occur along its western shore.

The 22 km coastline is bordered by a continuous belt of fringing coral reefs with a fore reef slope that dips steeply into deep water. Coral reef area is 10.5 km^2^. These reefs are in virtually pristine condition, with high coral cover that locally exceeds 80%, and scarce macroalgae [37, 43]. Coral communities have been impacted by bleaching, presumably severely in 1998 [44] and moderately in 2011 [43], due to high positive temperature anomalies [45]. Fish biomass is remarkably high, reflected in the presence of sharks, large piscivores, herbivores and large schools of planktivores [37].

### Field observations

A scientific expedition composed of two legs was conducted at the onset of the cool season in 2013: from 30 April to 12 May (1^st^ leg) and from 3 to 13 June (2^nd^ leg). During this period, strong south-east trade winds restricted observations on the windward coasts, entailing that one third of the survey sites were located in the north-west side of the island and only 7 dives in the south (Table 1). In spite of difficult sea conditions, 30 sites scattered along Europa’s entire coastline were studied, among which 8 were visited 3 times or more (Fig 1).

**Table 1:** For each of the 7 shark species recorded at Europa Island in April-June 2013: Percentage of dives with shark observations, mean ± s.d. of shark counts per hour and frequency of shark sightings according to day-period and species distribution, in both cases with the total number of individuals within brackets: Scuba diving (in normal font), chance observations at sea surface (in bold) and in the lagoon (in italics).

Species	% of dives with sharks	Dive and sea surface observations									Lagoon	Total
		8–11 H	>11–14 H	>14–17 H	N	NE	E	S	W	NW		
*Carcharhinus albimarginatus*	25.4	0.23 ± 0.63 4 (7)	0.69 ± 1.01 8 (11)	0.38 ± 0.74 5 (8) + **1 (1)**	**1 (1)**	0.42 ± 1.16 2 (5)	0.64 ± 0.67 6 (7)	0.86 ± 0.90 4 (6)	0.89 ± 0.93 5 (8)	0	*0*	0.39 ± 0.78 17 (26) + **1 (1)**
*Carcharhinus amblyrhynchos*	3.0	0	0.12 ± 0.34 2 (2)	0	0	0.08 ± 0.29 1 (1)	0.09 ± 030 1 (1)	0	0	0	0	0.03 ± 0.17 2 (2)
*Carcharhinus galapagensis*	28.4	0.70 ± 1.39 8 (21)	0.94 ± 1.57 6 (15) + **1 (1)**	0.81 ± 2.25 5 (17)	0	0.25 ± 0.62 2 (3)	1.00 ± 1.48 4 (11)	4.00 ± 3.16 7 (28)	1.11 ± 1.36 5 (10)	0.04 ± 0.21 1 (1) + **1 (1)**	*0*	0.79 ± 1.72 19 (53) + **1 (1)**
*Carcharhinus melanopterus*	1.5	0.03 ± 0.18 1 (1)	0	0	0	0	0	0	0	0.04 ± 0.21 1 (1)	*4*.*50 ± 5*.*07 4 (18)*	0.01 ± 0.12 1 (1) + *4 (18)*
*Galeocerdo cuvier*	1.5	0.03 ± 0.18 1 (1)	0	0	0	0	0.09 ± 0.30 1 (1)	0	0	0	*0*	0.01 ± 0.12 1 (1)
*Sphyrna lewini*	19.4	1.73 ± 5.08 7 (52) + **4 (41)**	3.56 ± 12.45 3 (57)	3.29 ± 9.09 3 (69) + **2 (151)**	**1 (150)**	0.42 ± 1.16 2 (5)	1.45 ± 2.94 3 (16)	10.00 ± 19.14 2 (70)	3.91 ± 9.47 1 (1)	10.00 ± 20.41 5 (86) + **5 (42)**	*0*	2.66 ± 8.51 13 (178) + **6 (192)**
*Sphyrna mokarran*	3.0	0	0.12 ± 0.34 2 (2)	0	0	0	0.08 ± 0.29 1 (1)	0.014 ± 0.41 1 (1)	0	0	*0*	0.03 ± 0.17 2 (2)
Total without *S*. *lewini*	31.3	0.74 ± 1.21 9 (30)	1.54 ± 2.33 7 (30) + **1 (1)**	1.22 ± 2.94 8 (25) + **1 (1)**	**1 (1)**	0.90 ± 1.60 4 (9)	1.88 ± 2.47 5 (21)	4.40 ± 4.51 5 (35)	1.50 ± 1.20 6 (18)	0.06 ± 0.24 2 (2) + **1 (1)**	*4*.*50 ± 5*.*07 4 (18)*	3.93 ± 9.27 22 (85) + **2 (2)** + *4 (18)*
Total	50.7	2.73 ± 5.98 16 (82) + **4 (41)**	5.44 ± 13.70 10 (87) + **1 (1)**	4.48 ± 9.31 8 (94) + **2 (152)**	**1 (151)**	1.17 ± 1.70 6 (14)	3.36 ± 3.59 8 (37)	15.00 ± 20.28 7 (105)	2.11 ± 2.15 7 (19)	4.00 ± 9.59 6 (88) + **6 (43)**	*4*.*50 ± 5*.*07 4 (18)*	6.09 ± 20.07 45 (475)
Number of field observations	67	30 + **4**	16 + **1**	21 + **2**	6 + **1**	12	11	7	9	22 + **6**	*5*	79
Number of species	7	5	5	3	2	4	6	4	3	3	*1*	7

Sharks were surveyed while scuba diving on coral reefs by 6 observers working in pairs. Each pair was composed of one scientist working on benthos accompanied by a professional diver in charge of surveilling sharks. The two dive directors were concomitantly diving in the surroundings, making shark observations and filming. Features, such as direction of movement and size of animals were mentally noted, and compared retrospectively among divers immediately after each dive, to avoid double counting of individuals [6]. A standard scientific dive lasted ca. one hour. A total of 67 1-hour scuba dives with random swim between 5 and 35 m depth were made mostly at constant depth: 30 in the morning between 8:01 and 11:00 hrs, 16 around mid-day between 11:01 and 14:00 hrs, and 21 in the afternoon between 14:01 and 17:00 hrs (Table 1). Water clarity is high in Europa with horizontal visibility of approximately 30 m.

Furthermore, 7 observations at sea surface were made while snorkeling (4 during the first leg, 3 during the second leg), and 4 observations (2 per leg) at sea-surface out of 5 visits in the lagoon from the dinghy between 16:30 and 17:00 hrs. Hence, scuba diving or snorkeling above the reef slopes and lagoon observations totaled 79 observation events.

Specimens were photographed and, or video (GoPro HERO2) recorded for subsequent confirmation of identification by Bernard Séret. Moreover one record from an underwater video on April 9^th^, 2019 (Thomas Claverie, pers. comm.) was also included as it showed an additional species. In all cases, no attracting devices were used. In the lagoon, size of individuals was visually estimated at ~5 m distance from dinghy to the nearest 10 cm with reference to bottom features such as individual coral colonies.

### Data analyses

Chi-squared (χ^2^) tests were used to test whether the probability distributions of shark occurrence according to day-period and location differed from random. Day periods were morning 8:01–11:00 hrs, mid-day 11:01–14:00 hrs and afternoon 14:01–17:00 hrs. Sites were pooled into 6 groups: north, northeast, east, south, west / west-northwest, and northwest. Tests were performed for overall shark presence and abundance, and at species level for the three most observed species. Data were not organized according to depth strata.

### Literature review

Shark diversity at Europa was assessed based on detailed review of the scientific and gray literature to identify previous reports of sharks in Europa, situate our records in this evaluation, and provide a species list for the island (Table 2). Some identifications have been verified on a wildlife documentary and its behind-the-scenes videos [31], for which the use of baits was reported. Other documentaries of Europa have been viewed but did not provide additional information.

**Table 2:** List of shark species recorded at Europa Island.

Shark species recorded from Europa Island	English name according to FAO	Fourmanoir 1952	Fourmanoir 1961	Maugé 1966	Probst 1998	Bertrand *et al*. 2001	Bourjea *et al*. 2006	SOS Foundation 2010	Séret *et al*. 2011	Clarke *et al*. 2012	Fricke *et al*. 2013	Chabanet *et al*. 2016	Present paper	Quotation total
*Carcharhinus albimarginatus* (Rüppell, 1837)	Silvertip shark		**+**	**+**	**+**	**+**		**+**	**+**	**+**	**+**	**+**	**+**	10
*Carcharhinus amblyrhynchos* (Bleeker, 1856)	Gray reef shark					**+**		**+**			**+**		**+**	4
*Carcharhinus brevipinna* (Müller & Henle, 1839)	Spinner shark		**+**	**+**										2
*Carcharhinus falciformis* (Bibron in Müller & Henle, 1839)	Silky shark							**+**						1
*Carcharhinus galapagensis* (Snodgrass & Heller, 1905)	Galapagos shark							**+**		**+**			**+**	3
*Carcharhinus longimanus* (Poey, 1861)	Oceanic whitetip shark				**+**									1
*Carcharhinus melanopterus* (Quoy & Gaimard, 1824)	Blacktip reef shark	**+**		**+**	**+**	**+**		**+**	**+**	**+**	**+**		**+**	9
*Carcharhinus obscurus* (Lesueur, 1818)	Dusky shark	**+**				**+**								2
*Galeocerdo cuvier* (Péron & Lesueur in Lesueur, 1822)	Tiger shark	**+**		**+**	**+**	**+**		**+**			**+**		**+**	7
*Negaprion acutidens* (Rüppell, 1837)	Sicklefin lemon shark				**+**		**+**	**+**						3
*Triaenodon obesus* (Rüppell, 1837)	Whitetip reef shark							**+**			**+**			2
*Sphyrna lewini* (Griffith & Smith, 1834)	Scalloped hammerhead					**+**		**+**	**+**	**+**	**+**	**+**	**+**	7
*Sphyrna mokarran* (Rüppell, 1837)	Great hammerhead					**+**				**+**	**+**		**+**	4
*Odontaspis ferox* (Risso, 1810)	Smalltooth sand tiger shark					**+**								1
*Rhincodon typus* Smith, 1828	Whale Shark												**+**	1
**15 species**		3	2	4	5	8	1	9	3	5	7	2	8	
Shark species likely to occur in the Europa EEZ														
*Carcharodon carcharias* (Linnaeus, 1758)	Great white shark	Ocearch tracking maps												
*Prionace glauca* (Linnaeus, 1758)	Blue shark	IOTC maps of catches												
*Isurus oxyrinchus* Rafinesque, 1810	Shortfin mako	IOTC maps of catches												
*Alopias* spp.	Thresher sharks	IOTC maps of catches												

Data from literature and present study. Fricke *et al*. [36] compiled previous records.

## Results

**Fig 2:**
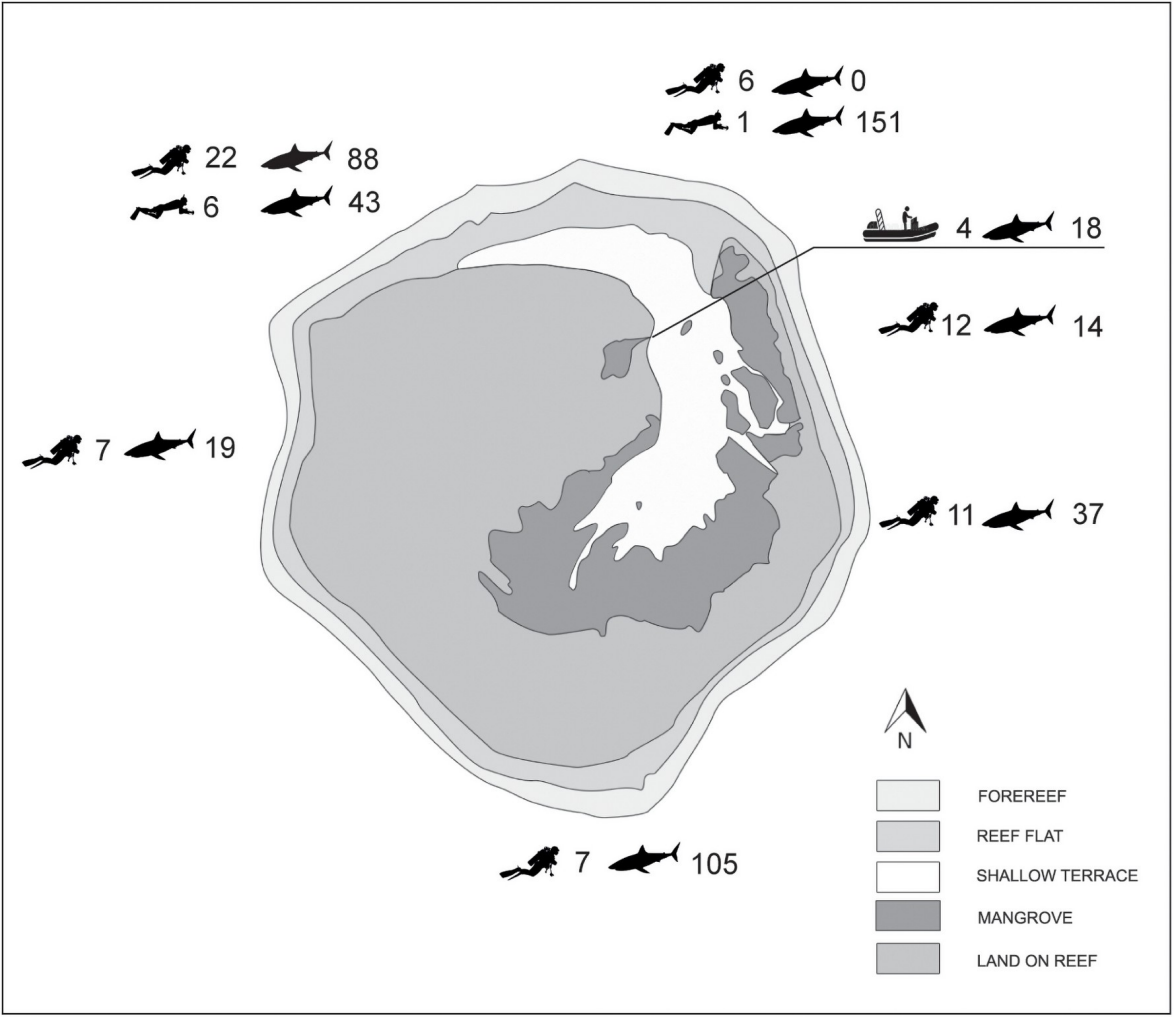
Number of field observations while diving, snorkeling, or from a dinghy in the lagoon, with corresponding number of sharks observed. Basemap adapted from https://eol.jsc.nasa.gov/SearchPhotos/photo.pl?mission=ISS005&roll=E&frame=9408 using Adobe Illustrator; Shapefile for diver retrieved from https://fr.depositphotos.com/1807037/stock-illustration-scuba-diving-silhouettes.html; for shark from https://pixabay.com/fr/vectors/animal-requin-silhouette-3364833/; and for boat from https://fr.dreamstime.com/bateau-icône-moteur-élément-d-transport-l-eau-des-applis-mobiles-concept-web-détaillé-peut-être-utilisé-image131545556.

In a period of 24 days, 475 individual sharks were counted during 79 observation events (Table 1 and Fig 2). A total of 263 sharks were observed during half of the 67 scientific dives performed between 6 and 35 m depth (34 dives with sharks and 33 without). The species most encountered were *C*. *galapagensis* (28.4% of the dives), *C*. *albimarginatus* (25.4) and *S*. *lewini* (19.5); the other four species (*C*. *amblyrhynchos* [3%], *S*. *mokarran* [3%], *C*. *melanopterus* [1.5%], *G*. *cuvier* [1.5%]) were only sporadically observed (Table 2). The most abundant species was *S*. *lewini* with a total of 178 individuals tallied while diving (2.66 ind. ± 8.51 s.d. per dive), and 192 individuals while snorkeling. One school, extending from the sea surface to ~15 m depth, was estimated at 150 individuals (S1 File) and smaller schools numbering 50, 30 and 20 individuals respectively contributed to this high abundance (almost 80% of the total shark abundance, 2/3 of the sharks observed while diving). Next in abundance while diving were *C*. *galapagensis* (53 ind., 20% of total, 0.79 ind. ± 1.72 s.d. per dive, S2 and S4 Files), then *C*. *albimarginatus* (26 ind., 10% of total, 0.39 ± 0.78, S3 and S4 Files), *C*. *amblyrhynchos* (2 ind., 0.03 ± 0.17, S5 File), two *S*. *mokarran* (0.03 ± 0.17), one *G*. *cuvier* and one *C*. *melanopterus* (both 0.01 ± 0.12). The highest daily number of sharks observed with scuba (May 4^th^) was 77 individuals belonging to 4 species (S1 Table).

The probability of observing sharks, including or not observations at sea surface, did not vary with time of the day (χ^2^ = 5.49, df = 2, p>0.05; χ^2^ = 5.31, df = 2, p>0.05), but the number of individuals observed were higher at midday and during afternoon compared to the morning period (χ^2^ = 22.03, df = 2, p<0.001; Table 1). If the observations of *S*. *lewini* from sea surface were included, the number of individuals observed in the afternoon was 2–3 times that observed at other periods of day, due in particular to the large school of *S*. *lewini* with large females appearing at sea surface at that time. When surface observations and those of *S*. *lewini* were excluded, most sharks were seen around noon. *C*. *albimarginatus* was seen more frequently (χ^2^ = 8.54, df = 2, p<0.05; χ^2^ = 7.88, df = 2, p<0.05) and in higher numbers (χ^2^ = 10.81, df = 2, p<0.01; χ^2^ = 9.58, df = 2, p<0.01) during the midday period (with or without observations from sea surface). *C*. *galapagensis* was observed while diving with equal probability and numbers regardless of the time of day (χ^2^ = 4.81, df = 2, p>0.05; χ^2^ = 2.93, df = 2, p>0.1), but numbers were slightly higher at midday when surface observations were included (χ^2^ = 7.62, df = 2, p<0.05). *S*. *lewini* was present any time of day (χ^2^ = 4.54, df = 2, p>0.1 and χ^2^ = 2.15, df = 2, p>0.25 respectively for all observations on the reef slopes, or during dives only). Finally, while diving the number of individuals at midday and in the afternoon were significantly higher than in the morning (χ^2^ = 55.40, df = 2, p<0.001).

*C*. *albimarginatus* and *C*. *galapagensis* were most frequently observed and in higher numbers off the eastern, southern, and western shores of Europa (Table 1; χ^2^ = 16.86 / 16.30, df = 5, p<0.01 and χ^2^ = 23.54 / 21.73, df = 5, p<0.001 for the first species, and χ^2^ = 17.41 / 16.69, df = 5, p<0.01 and χ^2^ = 58.59 / 55.28, df = 5, p<0.001 for the second one, including or not the surface observations), suggesting that both species prefer exposed habitats above calmer waters. *S*. *lewini* was observed all around the island with equal probability (χ^2^ = 6.38 / 4.48, df = 5, p>0.1, including or not the snorkeling observations) but numbers of individuals observed during dives were significantly higher in the south and in the northwest (χ^2^ = 300.00, df = 5, p<0.001), and higher in the north when including the surface observations (χ^2^ = 157.91, df = 5, p<0.001). Uncommon species such as *C*. *amblyrhynchos*, *G*. *cuvier* and *S*. *mokarran* were observed on the east coast but their very low occurrence and abundance prevent any generalization. All three species contribute to higher shark diversity at the island’s east point. Overall, the probability to cross paths with sharks while diving or also snorkeling was slightly higher in the south, west and east (χ^2^ = 16.60 / 14.08, df = 5, p<0.05) while their abundance was highest in the south (in the north when taking into account the large school of *S*. *lewini* at sea surface; χ^2^ = 160.24 / 339.08, df = 5, p<0.01).

Whereas *C*. *melanopterus* was observed only once on the coral reef slopes, west to the lagoon sill (Table 1), it was the only species seen inside in the lagoon and in a mangrove creek (18 ind. for 4 visits, only one visit without shark). There was a noticeable difference in estimated size among individuals of *C*. *melanopterus* according to their location, with smaller individuals of ~0.40 m length observed at the entrance of a mangrove creek at the western shore (stations 49 and 61) and bigger ones of 0.80–1 m length in a channel between an islet and the mangroves on the lagoon’s east side (station 32).

Overall, 7 shark species were documented (Table 2). Besides our own observations, one *Rhincodon typus* recorded on an underwater video taken in 2019 was added to our species list (Table 2). The present shark diversity was compared to previous records at Europa. Since 1952 [26], only 10 studies were reported. Half of them were gray literature and provided three of the 4 single occurrences that were all chance observationse.g.. The present study contributed one out of 4 single occurrences.

## Discussion

To present, a total of 15 shark species have been recorded at Europa, 8 of which are reported in the present paper (Table 2). Among these, the whale shark witnessed on the underwater video of 2019 is a new record.

In spite of the almost pristine condition of the marine habitats and abundant marine life, and high fish biomass [37] that should be suitable to support abundant populations of apex and meso-predators, the shark diversity at Europa is relatively low compared to that in neighboring areas (Table 3). This is similar to the situation at the other Eparses islands. The ‘deficiency’ might be due to the lack of observations and systematic studies on this group of fishes. Moreover compared to the number of shark species in the Mozambique Channel, or worldwide, records from the Eparses Islands are constrained, in particular because no inventory of deep-water species has ever been conducted.

**Table 3:** Number of shark species in the Eparses Islands compared to neighboring areas, in relation to some geomorphological parameters.

Location	Number of shark species	Number of coral reef- associated shark species	% of coral reef-associated sharks	Coral reef area (km^2^)	Lagoon area (km^2^)	ZEE area (km^2^)	Coast line (km)
Worldwide	540^1^						
South Africa	108^2^	19^2^	18			1,068,659	2798
Mozambique	67^2^	17^2^	25			578,986	2470
Madagascar	76^3^	19^3^	25			1,225,259	4828
Seychelles	38^2^	12^2^	32	1690		1,400,000	491
Aldabra	10^4^	10^4^	100		182		
Mayotte	26^1^	9^1^	35	413	984	63,000	135
Comoros	17^2^	8^2^	47	430	175	165,752	340
Juan de Nova	11^1^	11^1^	100	43	163	61,050	11
Glorieuses	7^1^	7^1^	100	27	170	285,000	9
Bassas da India	7^1^	7^1^	100	49	47	123,700	35
Europa	15^5^	12^5^	100	11	7	127,300	22
Agulhas current (ecosystem)	63^2^	14^2^	22				

^1^: BS unpublished data.

^2^: data from Fishbase (in March 2019).

^3^: [46].

^4^: [47].

^5^: present study.

Graham *et al*. [6] showed that shark populations declined in remote atolls of the Chagos Archipelago where the number of sharks observed per scientific dive collapsed from 4.2 in the 1970’s to 0.4 in 2006, representing a loss of 90%, most likely due to poaching. Between 1996 and 2012 *C*. *amblyrhynchos* largely dominated the shark assemblage on the outer reef slopes. Contribution of *C*. *albimaginatus* increased from 2001, reached almost 30% in 2006, decreased 4 to 6 years later but remained higher than in 1996. In 2012, the year before our observations in Europa, *C*. *melanopterus*, and *T*. *obesus* were again represented in Chagos after being absent from counts in 2006 and 2010, together with *C*. *amblyrhynchos* and *C*. *albimaginatus* in addition to *Nebrius ferrugineus* [19]. In 1977–1978 Stevens [47] fished at the Aldabra Atoll 1317 sharks comprising 9 species. At that time the most common shark was *C*. *melanopterus* (1046 individuals), followed by *Negaprions acutidens* (155 ind.), *C*. *albimaginatus* (52 ind.) and *C*. *amblyrhynchos* (as *C*. *wheeleri*, 41 ind.). The abundance of *C*. *melanopterus* varied from 19 to 198 ind. km^-2^ (mean 85 ind. km^-2^) according to reef geomorphology (lagoon, passes, reef flat, reef edge). More recently Friedlander *et al*. [48] noted that sharks were rare in the archipelago and occurred only at Aldabra where the presence of Seychelles Islands Foundation staff may dissuade poachers targeting shark fins. *C*. *melanopterus* represented only 3% of the observations versus 12% for *C*. *amblyrhynchos*; a shift in the composition of shark populations between both periods is plausible.

The numbers of sharks considered as coral reef-associated species are of the same order of magnitude, varying from 7 to 19 in the neighboring countries. However, they represent important differences when expressed as a percentage of the total number of shark species, varying from 18 to 100% (Table 3). Variations in coastal and lagoonal surfaces, and the state of knowledge of this particular fauna in these countries contribute to explaining these differences.

The shark fauna of Europa is composed of typical reef species like *C*. *melanopterus* and *T*. *obesus*, *N*. *acutidens*, coastal-pelagic species like *C*. *albimarginatus*, *C*. *galapagensis*, *C*. *amblyrhynchos*, *G*. *cuvier*, and *S*. *lewini*, and occasional visitors from open ocean, like *C*. *falciformis*, *C*. *longimanus* and *S*. *mokarran* (Table 4). Meso-predators like *C*. *galapagensis* [3], and to a lesser extent *C*. *melanopterus*, are rather frequent and abundant (Table 1). In contrast the apex predators *G*. *cuvier* and *S*. *mokarran* are rare, while *C*. *albimarginatus* (apex predator according to Frisch *et al*. [2]) is quite frequent. The occurrence of apex predators highlights the importance of Europa for conservation of sharks as they represent the most vulnerable functional role of marine food webs due to lack of functional redundancy [3].

**Table 4:** Habitats and geographical distributions of the shark species recorded from Europa Island.

Species	Habitat 1	Habitat 2	Depth range	Distribution
*Carcharhinus albimaginatus*	bentho-pelagic	reef-associated	0–800 m	tropical Indo-pacific
*Carcharhinus amblyrhynchos*	bentho-pelagic	reef-associated	0–140 m	tropical Indo-pacific
*Carcharhinus melanopterus*	bentho-pelagic	reef-associated	0–30 m	Indo-west-pacific
*Negaprion acutidens*	bentho-pelagic	reef-associated	0–30 m	Indo-west-pacific
*Triaenodon obesus*	bentho-pelagic	reef-associated	0–330 m	tropical Indo-pacific
*Carcharhinus brevipinna*	bentho-pelagic	coastal	0–75 m	circumtropical
*Carcharhinus galapagensis*	bentho-pelagic	coastal	0–180 m	circumtropical, patchy distribution
*Carcharhinus obscurus*	bentho-pelagic	coastal	0–400 m	circumglobal
*Galeocerdo cuvier*	bentho-pelagic	coastal & oceanic	0–140 m	circumtropical
*Sphyrna lewini*	bentho-pelagic	coastal & oceanic	0–1000 m	circumglobal
*Sphyrna mokarran*	bentho-pelagic	coastal & oceanic	0–180 m	circumglobal
*Carcharhinus falciformis*	pelagic	oceanic	0–500 m	circumtropical
*Carcharhinus longimanus*	pelagic	oceanic	0–152 m	circumtropical
*Odontaspis ferox*	bentho-pelagic	deep-water	0–500 m	circumglobal, patchy distribution

The only apex predator to be very abundant during this time of year is *S*. *lewini* that was often observed in aggregations of a few to several tens of individuals patrolling along the reef slopes (as reported in 2010 [33]), once forming a large school of ~150 individuals (S1 File). Schooling permits conspecifics to interact socially, including courtship displays, during the resting phase of their diel cycle rather than conferring protection from predation since smaller sharks are forced to the edge by the largest immature females [49]. We hypothesize that the vicinity of seamounts may favor *S*. *lewini* schooling in the area as this species is known to forage nightly down to 450 m depth, even exceeding 700 m, in the pelagic environment of offshore seamounts [50–53], which contributes to its ontogenetic development [54]. The Jaguar Seamount and Hall Tablemount lie respectively 100 and 140 km northeast Europa, and at 430–600 m and 170–700 m depth [39], distance and depth fitting with the ability of this cruising fish [54]. These seamounts constitute topographic constraints to the anticyclonic eddies that propagate southward through the Mozambique Channel [55] and locally provide leeward areas offering protection from currents and resting places for sharks. The particular geomagnetic sensitivity of *S*. *lewini* [50] could enable this species to detect these volcanic seamounts [39] that are known to have a magnetic effect [40]. Interestingly the SOS Foundation [33] in their survey conducted in July did not encounter any *S*. *lewini* at Bassas da India whereas they counted 55 individuals at Europa (mostly gathered in a school of 35 individuals) some days earlier. These time-restricted observations could suggest that this species does not cruise at Bassas da India in winter or have migrated already, a suggestion that should be explored by further investigations. Also this species was shown to display site fidelity to the tiny Saint Peter and Saint Paul Archipelago during a 120-day monitoring period [53], it could stay for some months within the cluster of seamounts. The travelling of *S*. *lewini* within the seamount archipelago together with potential site fidelity to the Europa—Hall Tablemount—Jaguar Seamount—Bassas da India cluster would be of great significance for the management of the area by the TAAF, and conservation of sharks and marine apex predators.

The inner lagoon of Europa is known to be a nursery for *C*. *melanopterus* as groups of young individuals are regularly observed moving in and out with the tides [26]. *C*. *galapagensis* possibly uses the lagoon of Bassas da India as a nursery [33, 56]. In this region the Tugela Bank lying north of KwaZulu-Natal along the south-African coast is known to be a nursery ground for *S*. *lewini* [57] as is Nosy Lava Island, in the Malagasy Mitsio Archipelago where this species used to be heavily fished (Jean-Bernard Galvès, pers. comm.). Since nurseries are crucial for maintaining fish populations, the occurrence of shark nurseries both in Bassas da India and Europa lagoons is an additional compelling justification for targeted shark conservation by the TAAF [56].

**Fig 3:**
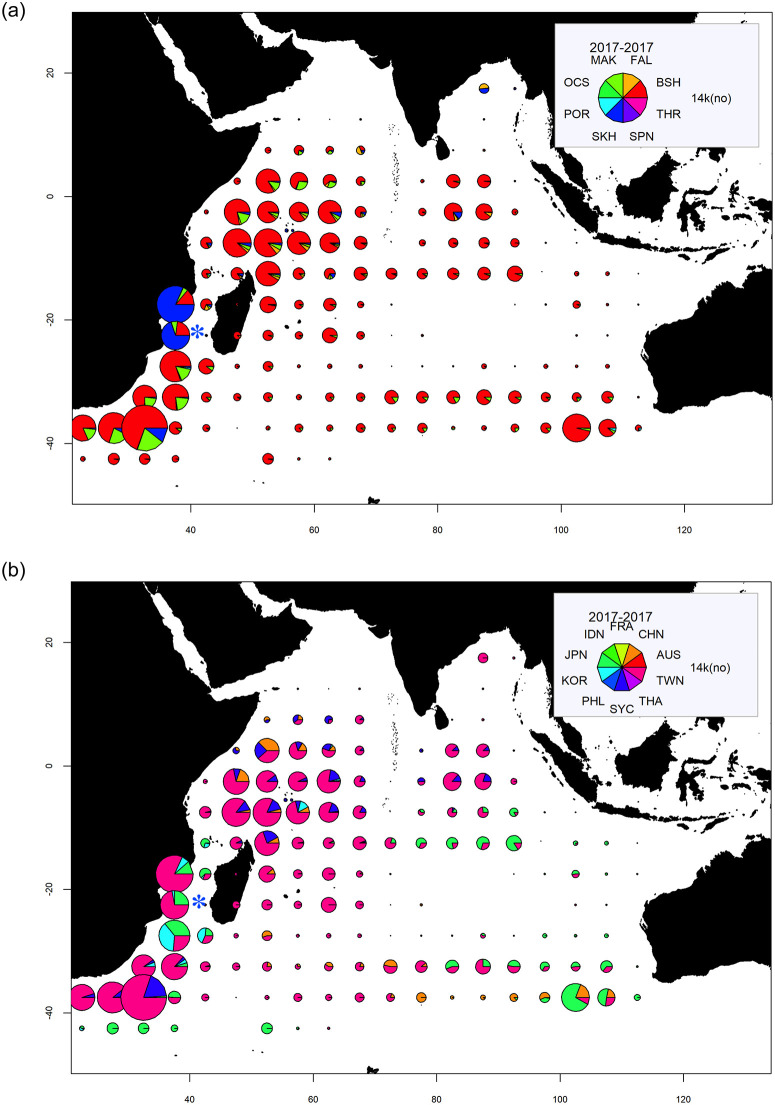
Maps showing the catches (total numbers) of sharks, by species and fleets in 2017 in the Indian Ocean. Reprinted from document IOTC -2018-WPEB14-07 under CC BY license, with permission from the Indian Ocean Tuna Commission, original copyright 2021. MAK: *Isurus oxyrhynchus*; FAL: *Carcharhinus falciformis*; BSH: *Prionace glauca*; THR: *Alopias* spp.; SKH: non-identified sharks; POR: *Lamna nasus*; OCS: *Carcharhinus longimanus*; SPN: *Sphyrna* spp. FRA: European Union France; CHN: China; AUS: Australia; RWN: Taiwan Province of China; THA: Thailand; SYC: Seychelles; PHL: Philippines; KOR: South Korea; JPN: Japan; IDN: Indonesia. Blue star: study site.

*C*. *carcharias* has never been recorded from Europa, but 12 records are known from southwestern Madagascar [58] off Morombé and especially the surroundings of Toliara, therefore about 300 km from Europa (Fig 1). Individuals tagged in South Africa have travelled in the southern Mozambique Channel as shown by the Ocearch Tracker (https://www.ocearch.org/tracker/). All these observations strongly support that *C*. *carcharias* likely cross the Europa’s ZEE during its migrations. Large pelagic sharks are commonly caught in the Mozambique Channel, in the vicinity of the Eparses Islands, including Europa as shown in maps of shark catches by tuna fleets ([59], Fig 3). These sharks should potentially be added to the faunal list (Table 2).

Aggregations of the whale shark *R*. *typus* are seasonally observed off the Mozambique coasts and in the northern and central Mozambique Channel [60, 61] where they are used by the purse-seiners as “natural aggregating devices”, mostly off Juan de Nova (*cf*. IOTC statistical data). One small female was reported from Bassas da India on April 8^th^, 2011 [34]. The presence of this species both at Europa (present video witness on April 9^th^, 2019) and close-by atoll early April contributes to the knowledge of the migration route of *R*. *typus* in the southern Mozambique Channel, providing relevant information since this species has been poorly reported in this part of the channel [60, 62]. One female satellite tagged off Nosy Be (Northwest Madagascar) swam to the southern edge of the Mozambique Channel, staying for a while in the southeast of Europa, and returned to the Nosy Be area [60]. Another female satellite tagged off the coast of southern Mozambique (Praia do Tofo) crossed the Mozambique Channel, bypassed the southern tip of Madagascar and reached the southeastern coast of Madagascar [63]. More tagging is needed to unravel the different routes of migration of *R*. *typus* across the southern Mozambique Channel and assess a possible role of Bassas da India and Europa, potentially along with Hall Tablemount and Jaguar Seamount, as feeding and eventually resting sites. This would further contribute to trace the southern limit of the distribution of this species.

The records presented here are in agreement with the collective memory of the wealth of sharks in Europa’s waters [64, 65]. In early 60’s, Goudeau [64] mentioned that sharks could be caught anywhere and readily near shore, in highest abundance in the lagoon, but no information on the species observed was provided. However, with the passing of years, our tally of 475 sharks at Europa within 24 days in April-June 2013 seems to have become remarkable. In comparison, during an expedition in the same period in 2018, including 8 observers and ~50 dives, relatively few sharks were encountered: *S*. *lewini* regularly, occasionally *C*. *albimarginatus*, *C*. *melanopterus* and *T*. *obesus*, plus one *G*. *cuvier*, and unexpectedly only one *C*. *galapagensis* (H. Bruggemann, pers. comm.). Also, during 3 weeks diving along the northern coast of Europa in December 2018, no *C*. *galapagensis* was seen. Instead, one *C*. *albimarginatus*, one *N*. *acutidens*, one *T*. *obesus*, approximately 15 *C*. *melanopterus*, and several small (2–3 ind.) groups and one larger school of 17 ind. of *S*. *lewini* were observed. Within 4 days in April 2019, apart from the whale shark filmed in the water column at ca. 35 m depth, only few *C*. *melanopterus* and only one *C*. *albimarginatus* were seen, in spite of multiple diving observers. In almost 60 years after Goudeau’s report [64], sharks were very rarely seen from the shore (MMM G, pers. obs.). Notably sharks were no longer present at the same sites where they were abundant in 2013 (Ricardo Beldade, pers. comm.). The decrease in shark numbers with time at Europa could well testify the ongoing decline of shark populations in the southwestern Indian region. Indeed, Europa is situated in an area of important industrial fisheries (targeting mostly tunas and billfishes) whose main by-catches are sharks. These legal fisheries are managed by the Indian Ocean Tuna Commission, with specific rules aiming to reduce their impact on shark populations. However, there are evidences of poaching and illegal fisheries in waters around Europa. Their impact is unknown but likely to be important when these fisheries target sharks. Control measures should be reinforced to curb the damages done by these fisheries.

## Conclusion

Overall, with the occurrence of several species of apex predators in addition to that of *R*. *typus*, large schools of *S*. *lewini*, a nursery of *C*. *melanopterus* and fair numbers of reef sharks, Europa’s sharks constitute a significant reservoir of biodiversity which contributes to preserving the functioning of the ecosystem. Our observations highlight the relevance of Europa Island for shark conservation in the Southern Mozambique Channel, and for which reinforcement of the surveillance capacities of the TAAF in the EEZ of both Europa and Bassas da India is a vital necessity. It might be that our observations represent one of the last testimonies of high shark abundance at Europa.

The present assessment of shark diversity at Europa based on our field observations and a literature search should be complemented with other techniques, such as BRUV systems [e.g. 10, 33, 35], and especially environmental DNA analyses [66]. While the molecular reference databases for shark exist [67], they still need to be completed to improve detection at species level [66]. Such surveys should be performed repeatedly to describe seasonal and annual variability. Also the bathyal zone around Europa should be explored, as numerous deep-sea sharks have been reported in the Mozambique Channel, including new species.

Our observations of schools of *S*. *lewini* and individuals of *R*. *typus* give rise to many questions for which studies including tagging with acoustic transmitters [e.g. 68] and genetic analyses [69, 70] will provide information about (i) their residence time at Europa with eventual site-fidelity, migration paths, and eventually including deep incursions at Hall Table mount and Jaguar Seamount, as well as (ii) their population structure, in particular in the Mozambique Channel. This knowledge is needed for adjusting to its behavior the conservation of these poorly known and endangered species as recorded on the CITES list. Such studies should also concern *C*. *galapagensis* and *C*. *melanopterus* which have nurseries respectively in Bassas da India and Europa. Yet conservation of adults is as crucial as juveniles for organisms like sharks, whose life histories are characterized by late reproduction age, low fecundity and large body size. Nowadays, the southwest Indian Ocean Fisheries commission (SWIOFC) of the FAO manages marine resources in the region [71] where small-scale fisheries are underreported [72, 73], aiming to secure sustainable fisheries and reduce illegal fishing. Sharks should benefit from this conservation action.

## Supporting information

S1 File *Sphyrna lewini*. School of ~150 individuals of *Sphyrna lewini* at Europa Island (site 67).
(MP4)

S2 File *Carcharhinus galapagensis*. *Carcharhinus galapagensis* at Europa Island.
(MP4)

S3 File *Carcharhinus albimarginatus*. *Carcharhinus albimarginatus* at Europa Island.
(MP4)

S4 File *Carcharhinus albimarginatus* and *C*. *galapagensis*. *Carcharhinus albimarginatus* and *C*. *galapagensis* at Europa Island.
(MP4)

S5 File *Carcharhinus amblyrhynchos*. *Carcharhinus amblyrhynchos* at Europa Island.
(MP4)

S1 Table Dataset. Details of the 79 observations events of sharks in April-June 2013 at Europa Island.
(XLSX)
